# Pyorrhea Alveolaris

**Published:** 1902-07-15

**Authors:** E. B. Newell


### PYORRHEA ALVEOLARIS.

BY E. B. NEWELL, D.D.S.

Read at the Meeting of the Michigan State Dental Association, at Grand Rapids, June 9, 1902.

At the request of the honored president of our local society I have endeavored to prepare a few words on the subject of "Pyorrhea Alveolaris", which I hope will be of interest, even if they should excite little or no comment.

During the closing years of the last century came great advancement and higher ideals, not only in general science, but in the art of dentistry. Before this time it consisted of a limited number of crude operations performed by those who had received their instruction as apprentices, and oftimes by only the self educated.

Their knowledge was meager, as pathology and therapeutics was practically unknown, and their system of treatment of diseased conditions was usually that laid down by the medical profession; but by hard and conscientious work they added each day a little more and a little more, until now, with the opening of the new century, they have unraveled for us to a large degree, many of the causes of dental diseases.

The relationship of dental disorders and diseased conditions has been compared with the normal, thus adding greatly to our working knowledge and putting us well up the steps of possible achievement.

If so much has been done for us, why should we not only do an equal amount, but far more, so that early in the present century will come an era that will forever stand out as a bright spot in the history of our chosen profession.

That which we should most desire to know, and must know, in order to accomplish what we hope to, and be of greatest service to our fellow beings, is the cause of the various deviations from the normal.

If we would know how to cope with such a destructive and persistant disease, as I have been asked to briefly take up to-day, we must look for the origin.

Is it contracted as a result of our mode of living, or were we originally born diseased? Is it the result of perverted appetites and uncontroled passions, thus throwing us out of harmony with nature, or is there some pre-existing weakness which causes the disintegration and loss of those tissues which are so essential for a perfect denture?

All of these things we must battle with and conquer, or our work will be for nothing, and the world will be no better from the fact that we have lived.

Along with us we have the medical profession in whose ranks are thousands of brilliant men, and each day they add a little to our knowledge, and thus assist us. Are we to be out-done and left behind by them?

Each and every one of us must make his individual answer and act accordingly, by personal research and by co-operation with others, so that before long we may be able to say to our patients, "Yes, we can successfully treat you for Pyorrhea." It is not an impossibility to cure it now a days, as our knowledge of its causes and our present modes of treatment certainly do bring about most satisfactory results.

Each man may have his theory as to the best course to follow, and for a time his system of treatment may be so extensively practiced that it may even be considered a fad and consequently something of short life; but if he is careful and conscientious in his study, I believe that the condition of helplessness on our part will soon be a thing entirely of the past.

To-day the average practitioner practically refuses to even try to cure pyorrhea. This is a mistake, at least, for the younger men. It is true the young man is apt to err in judgment, due to lack of experience, but the patient will expect him to do more difficult things than the older men of the profession, and he must risk operations they would not attempt, although he would be blamed if he should fail.

The older men are not likely to be pioneers in the new field of surgery, so they adopt the conservative, or "let alone" policy, which up to a recent date seems to have been regarded as the proper course to follow.

This being the case it behooves us to not only make ourselves acquainted with the writings and teachings of the previous generations, but to personally experiment with these conditions and add to our resources that which will make us the victors.

The various causes of this most discouraging disease with which we have to deal have often been given as degeneration and disintegration of the various structures, due to excess of uric acid or rheumatism. This, no doubt, is true to a great extent, but it is not enough nor are we satisfied with it.

The word "itself" means but little, as it refers to but one of the symptoms common to the several varieties of disease of the peridental membrane, i. e., pus in the sockets of the teeth; while in reality many other distinctive features exist, such as progressive loosening of the roots, attended by complete loss of the retentive structures and alveolar walls, resulting in a cessation of all difficulty upon the removal of the affected teeth.

We find that this disorder evidences itself most commonly in the adult, although in exceptional cases it appears even during childhood.

There have been many attempts to classify all cases of tooth loss, characterized by the features named, under one head or class, based upon the pathogenesis of the disease, but they have failed, and in many cases done much to assist in the confusion which already existed as to its causes, prognosis and treatment.

We know that in every instance where pus formation is found, the pyogenic staphylococci and streptococci are attendants, but every effort to isolate a specific bacterium has signally failed. Each author quotes the other and their research is all indeterminate.

If we put aside the causes, both remote and direct, we may clinically divide the cases into three classes;

First.—Those associated with that form of gingivitis in which we find dark, hard, scaly annular calculi just under the margin of the gums.

Second.—Those cases in which gingivitis can hardly be detected, nor is there usually any deposit in the early stages, but the necrosis of the peridental membrane advances in such a manner that it has been designated, phagedenic pericementitis.

Third.—Cases in which the gum margin is apparently normal, but where the degeneration and necrosis of the pericementum, and also the deposits of calculi, occur at some lateral aspect of a root.

Each of these classes, if divided in this way, have some features in common, but they differ as to their causes, prognosis and method of treatment. If we take the first class we will note that pericemental degeneration apparently is a secondary feature, while in the second it is the distinguishing one; but in the third, only circumscribed portions of this membrane display the first evidences of the disease.

The predisposing causes of all of them are no doubt the diseases of suboxidation, but only in the last case can it be said that a gouty condition is the probable exciting cause.

The predisposing causes, both general and local, of the first class are those which bring about reduced vitality, such as misuse, disuse or overuse of the teeth, while the exciting causes are not only gingivitis, but deposits of calculi and infection of the already irritated tissues, while later on, looseness of the tooth, or death of the pulp may enter into it.

As to the symptoms of the disease they differ greatly during its progress. We commonly see the disorder after its first stages, i. e., we find the gum margin swollen, red and oedematous, all of which conditions vary with individuals and causes. Pronounced swelling and a purplish color of the gum is sometimes found, but ordinarily this is not the case. Later on if the gum margin is closely examined, dark green calculi are found on the root, although in some cases the calculi are formed without the morbid condition of the gum.

The caculi are no doubt the result of the uniting of the calcium salts in the saliva with the inflammatory exudates, and they continue to form, further encroaching upon the pericementum until the root is denuded. Usually the line of alveolar process recedes in proportion to that of the root denudation, and evidences of infection appear, as pus may be pressed from the gum margin. In the early stages the tooth is not sore unless pus is confined, but there is most always a perceptable looseness which later excites an inflammatory reaction in other parts, thus increasing both of these conditions.

At this time the pulp of the tooth is usually strangled, the result being apical pericementitis, which continues until complete atrophy of the affected membrane and the alveolar walls causes the offender to be cast out. The entire mouth is usually affected by this abnormal condition. The diagnosis of this form of pyorrhea is not often difficult. A careful examination will usually show that more than one tooth is affected. The upper incisors I have found most commonly attacked.

After the disease has fully evidenced itself there is little liability of a mistake in diagnosing this form of the trouble. The gum has a peculiar swollen appearance, deposits of calculi can easily be detected in the pockets, and pus oozes from the gingival margin, so that one would not be liable to confound this condition with any other dental disorder.

The normal line of the gum often recedes before there are any evidences of calculi and before any gingivitis can be noted, so that the gum festoon is completely destroyed.

At times it is quite difficult to determine which class of pyorrhea is present when the later stages are reached, but if the pockets are found to extend little beyond the deposits of calculi, it may be considered as a case of the first class, and the gingivitis keeps pace with the destructton of the pericementum.

I believe that it is possible to completely arrest this form of the disease, provided we can remove every source of irritation and the treatment would of course, consist of this first, followed by a general toning up of the tissues affected, and an improvement in the general health.

Very loose teeth should be carefully lashed together and splints made to hold them firm while they are being scaled and ground to articulate properly.

The ingenuity of the operator will ofttimes be taxed in the making of a splint of this kind, as each individual case presents entirely different conditions.

After the roots are made firm I usually carefully syringe the pockets with hydrogen dioxid, using a syringe with a fine point and considerable strength, so as to force the medicine deep under the gum line.

Of late I have used twenty-five percent pyrozone in the pockets and with splendid results.

The teeth should be thoroughly cleaned and every particle of deposit should be removed from the roots with carefully prepared scalers, made according to the operator's best judgment.

When pronounced swelling is encountered it is a good idea to pack the pockets with pellets of cotton saturated with ten percent solution of trichloracetic acid. When they are removed it will also give you a better view of the interior.

A very essential feature in the treatment is to have the patient use an antiseptic and an astringent mouth wash regularly at least twice a day, which you personally prescribe. Washes containing hydronaphtol, thymol, alcohol, glycerol, benzoic acid and many others I prescribe regularly.

The second class of cases which has so often been designated as phagedenic pericementitis, was first called so by Dr. Black, because of its peculiar destructiveness of the pericementum. This name in reality covers two classes or conditions of pyorrhea, i. e., the cases in which the destruction of the membrane is accompanied by deposits and inflammation, and those where it takes place without them.

It is a question with me whether or not it should be called pyorrhea, but because of the flow of pus it is convenient to classify it in this way.

Upon careful examination, degeneration, atrophy, and necrosis of the process and destruction of the pericementum are found, accompanied by deposits of calculi and pus.

Portions of the osseous tissue and the membrane may be completely destroyed without noting any inflammation.

As to the causes of this disorder it is quite difficult to determine, but everything points to heredity. Beside this, faulty occlusion or abuse of the teeth, predisposes the membranes surrounding them to destruction in this manner.

If all the teeth in a perfect denture were held firmly in their sockets and the articulation of each one with its antagonists could be equal and perfect, it is my opinion that even hereditary predisposition would not bring about the loss of many teeth in this way.

I believe that in the near future a distinctive germ will be found to be the cause of this disease, as heredity and all the predisposing and exciting causes I have heard of so far, do not explain nor do they satisfy.

The upper central incisors and the lingual portions of the upper molar roots I have found most subject to attacks, and oftimes a tooth will shift its position, even before any other evidences of the difficulty manifest themselves.

Soon after this it becomes quite loose, especially if the occlusion becomes faulty ; but the gums between the teeth most always recede before loosening takes place, showing a condition of atrophy of the edges of the alveolus.

If a pocket is examined it will be found to extend almost the entire length of the root, showing that the destruction of the membrane is rapid in one way and slow in another, as ofttimes I have found that a flat blade would work its way almost to the apex and yet it was difficult to insert it at first under the margin of the gum.

Pus most always appears before a pocket becomes very deep, but when pus is present the small nodular deposits are usually found on the root.

An abscess forms at the apex of the root, if the destruction of the pericementum continues to that portion and discharges through the pocket.

This disease is peculiar in that it is often found in mouths, the care of which has been rigidly looked after, and where no deposits are to be found, nor is there necessarily any indication of inflammation about the gum previous to its appearance. When one tooth is affected, as a rule it is not long before several others are attacked in the same manner.

The patient is usually of the bilious or sanguinary temperament and the teeth do not seem to contain many decayed places, in fact I have found in many cases where dental caries had been in evidence before any signs of pyorrhea appeared, that afterward the decay seemed to stop almost entirely.

This disease is a disintegration of the pericementum, which I do not believe is hereditary, but a predisposing weakness may exist, because of the fact that a parent was troubled with it.

In diagnosing a disease as phagadenic pericementitis, my first step is to consider the peculiar pockets and denuded roots. The temperament and age of the patient are important.

The moving of a tooth without apparent reason after the patient has reached the age of about thirty, most always points to this trouble.

Of late I have had several cases where it was quite difficult to determine whether or not apical abscess existed If an acute abscess exists the symptoms of acute abscess will help in determining, but if a chronic condition is found, matters are more complicated.

The condition of the pulp and the result of testing with hot and cold will often decide.

Metal fillings in the immediate vicinity of the pulp often enter into the matter.

One of the best ways to determine is to carefully examine the teeth on both sides of the offender and one will usually find evidences about the other teeth, unless it is a case of apical abscess.

This trouble, if well advanced, is decidedly discouraging, because it almost always returns unless constantly watched and treated with great care, but regularity of treatment I have found of the greatest importance, and that not only by the operator, but by the patient himself.

A thorough cleaning and the removal of all disturbing factors, such as deposits and necrosed tissues, is the first essential for a return of the part to health. If the disease is discovered early, a little regard for oral hygiene and the avoidance of misuse of the teeth will be of great assistance in the treatment.

As a rule, if the pulp of a tooth is removed at the first evidences of developing pyorrhea, the disease is arrested, and in my opinion this is good practice except in the upper incisors. The nourishment of the pulp seems to be transferred to the surrounding membranes of the tooth after the pulp is destroyed, thus increasing their powers of resistance.

After the careful removal of all irritating matter by the use of scalers, and not being so particular to avoid cutting the tissue surrounding the pockets as in the first case, except at the gnm line; a thorough sterilization is necessary, and the treatment may be continued similiar to that followed in the first class.

Cauterants are more essential in these cases, I have found, than in the other forms and to freely scrape the edges of the necrosed alveolus, after making an incision through the gum tissues, does wonderful good.

Those cauterants I have found most useful so far are: trichloracetic acid in ten percent solution, sulphate of copper carefully packed down in the pockets, protecting the mouth with a napkin, and a twenty percent solution of the iodide of zinc applied well under the margin of the gum. Lactic acid was recently suggested to me, but as yet I have had no results.

The third class, or gouty pericementitis is usually found in persons of an arthritic diathesis, and I am firmly convinced that this condition furnishes not only the predisposing, but the exciting causes of the disease. The deposits of calcium, which are usually attendants of the diseased area, exhibit a combined reaction of urates and calcium phosphate and act as exciting causes in the destruction of the predisposed membranes of the teeth.

The symptoms of this particular form depend upon the time or stage at which it is seen, as the case may often extend over a period of years, but if the history of the family is noted, gout is most always found.

Neuralgic pains are often felt about the jaws at night, and one or more teeth will shift position without evident local cause.

At first the tooth will respond decidedly to thermal changes, a general heightened sensitivity of the dental pulp existing, all of which usually disappears after a time. People suffering from this class of disorders are very subject to erosion and abrasion and the exposed dentin furnishes cause for reflex pains about the jaws. Later on a single tooth may become sore upon percussion and we may note that the gum overlying the apical portion of the root is affected by a varying inflammation in the deeper tissue, but the gum margin may remain unbroken, although the tooth is loosened as a result and usually remains so. The symptoms are similar to acute septic pericementitis at the apex of the root, although less marked. Infection is not alvays certain in these cases, but if so, and an incision is made into the swollen portion, a glarry mucus like discharge will take place, which may or may not contain pus. Under these conditions a mistake is often made in diagnosis and the operator drills into the tooth, which may not exhibit sensitivity until the instrument is plunged into a vital pulp, which shows that there is no connection between the inflammation, or suppuration, and the pulp.

If the abscess cavity is enlarged and examined carefully, the alveolar wall will be found absent, exposing the side of the root, upon which dark calculi can be seen.

If the patient is vigorously treated for gout, during the early stages when there is very little pus formation, the result is almost universally a rapid reduction of the inflammation.

When a person suffers from gout, I believe with others that it is due to the retention in the circulation of an excess of urates, a waste product, which acts as an irritant in different parts of the body, producing alteration of functions in many tissues and organs, but most often in the connective tissues. Uric acid is one of the animal poisons generated in the living tissues, and is an oxidation product of albumenous matter. There are several of these poisons depending upon the degree of oxidation, the last of which is urea, which is found to be freely soluble in the serum. This urea is thrown off by the kidneys as a product of nitrogenous waste if the kidneys are in a normal condition, but if the oxidation is faulty uric acid is formed instead, and we find it necessary to displace the sodium, to which it unites in the blood, with lithium, or potassium, which are much more soluble.

It has been demonstrated that if the leucocytes in the blood current are increased and the red corpuscles are diminished in proportion, there is a notable increase in uric acid, showing that a deficiency of oxygen is carried to the tissues. Sluggish vascular flow, caused by accumulated waste products, interfers greatly with oxidation, and adds to the causes of this difficulty. If such a condition exists it is probable that deposits of crystals of urates will take place where the currents are sluggish, such as in the articular cartilages of small joints, the vessels of which have already become rigid, as is usually the case with those troubled with this class of difficulties, thus reducing the vitality of the membranes and predisposing them to other attacks.

Now in reality the pericementum is a ligament, as well as a periosteum, thus making the union of a tooth with the alveolus a joint and subject to attacks in the way and for the same reasons, because it is a joint.

A proper amount of use of the teeth is necessary, or the nutrition of the pericementum will become sluggish, the same as in other joints, and as a result, subject to degeneration and a predisposition to all of this kind of troubles.

To successfully treat this abnormality I always find it necessary to improve the general as well as the local condition. The waste products should be washed out through the bowels and kidneys and the alkalinity of the blood should be increased. Continued use of lithia waters usually results satisfactorily, or five to ten grains of bitartrate of lithia each day disolved in drinking water. It is well to advise the patient to use white meat and fish in place of dark meat and beef. Open air and plenty of exercise of course are very beneficial, as they tend to increase the oxidising function.

A very good local treatment I have found is to follow along the same line as in treating the other forms, i. e., thorough cleaning of the teeth, removal of all deposits under the gums, by scaling and the use of solvents. The use of twenty-five percent pyrozone carried to the pockets on pellets of cotton is beneficial. "Philip's Milk of Magnesia" is splendid to neutralize conditions of acidity.

The contraction of loose tissue and the lessening of congestion, by the use of astringent and antiseptic washes, is necessary. Iodide of zinc, three grains to the ounce of water, is a good solution.

A soft rubber brush I think is the best for the patient to use, even after treatment has reduced the gums apparently to the normal, as the liability to irritation is greatly reduced. Observance of the rules of hygiene will not only help those who have already become victims of this disease, but will popularize a theory which may be the cause of preventing thousands of cases in the future generations.

Children inherit disease from their parents, and they may also inherit tendencies.

I think it advisable to replace a lost tooth with a bridge, unless those on either side are very loose, or the extent of the degeneration of the tissues is great, as it holds them in their proper position and does away with the necessity of a partial plate, which is usually very troublesome under these conditions. If bridge work is inserted the joints should be as near perfect as possible, and I am firmly convinced that the use of gutta percha as a cementing medium is far superior to phosphate of zinc, for many reasons.

There have been many new ideas advanced of late as to the causes of all of these forms of disease, such as hereditary syphilis, the excessive use of alcoholic liquors, shock to the nervous system, etc., but whatever truth there may be in them let us hope will soon be determined.

In conclusion it might be well for me to say that the aim of this paper has not been to bring forth new remedies, or methods of treatment, but to give an outline of what the subject means to me, and, if possible, to encourage others by pointing out the necessity of further research.

### DISCUSSION.

Dr. A. F. Rix: After listening to such an able and exhaustive paper as this, it would be superfluous for me to undertake to discuss the subject, especially to try to enlighten such men as the author of the paper. He has given a thorough history of the cause and a perfect diagnosis and prognosis as far as I know. I have treated this disease to the best of my ability and have sometimes cured and sometimes have not. My line of treatment has been much the same as that laid out in the paper but in addition to his methods I have used Robinson's remedy in the pockets, and also aromatic sulphuric acid, which materially assists in the removal of the calcareous deposits which form in connection with this disease.

Dr. Hoff: Like Dr. Rix, I think the essayist has given us the most concise and systematic classification of this disease and its treatment that I have ever heard. I do not think there has been a better paper read before any society in the last two years. Each one of us will have his peculiar methods of treating this disease, and I do not quite agree with the essayist in the use of some of the remedies which he advocates. His classification has been so well stated that it will enable us, if we understand the remedies which we use, to apply them more intelligently than before. The cases which he classifies under the first head are those where there is simply an irritation of the gum and other soft tissues, caused by calcareous deposits. He speaks of using in connection with these cases the escharotic or twenty-five percent solution of pyrozone.

Dr. Newell: I have not used that for a very long time, I heard through a Dr. Rowland that it was very beneficial in such cases, my use of it has not been very extensive.

Dr. Hoff: You also suggested trichloracetic acid which is another strong escharotic. I do not find that there is any indication for the use of such destructive escharotics for this condition, as they always destroy tissue and we have no tissue at this stage of the disease which we can afford to loose, it is all valuable, so I would take particular exception to such treatment. We may get good results because they are very powerful disinfectants, but I do not like to use them because they unnecessarily destroy the soft tissues. I prefer using milder remedies, disinfectants which are not escharotics, they may not be so strong, but will accomplish the desired results. A three percent solution of peroxid of hydrogen or five percent solution of lysol, are not escharotic and are sufficiently disinfectant. The lysol solution has an alkaline reaction and produces saponification of accumulations upon the teeth, I find this very desirable in the weak solutions. If I use considerable quantity I use less than a two percent solution, and inject with a large syringe which also aids in washing away the accumulations which occur in these pockets. This would be my line of treatment for disinfecting such cases.

In the next, or the phagedenic variety, there is more occasion for the use of an escharotic or caustic, but I have found in these cases also we are liable to create an irritation of all the surrounding tissue from excessive medication. I have induced this irritation by the use of trichloracetic acid, and also lactic acid. I am now treating a case where strong solution of pyrozone was used, it created so much irritation as to extend to the end of the root, and I have been over a week in trying to get the patient in a comfortable condition. It was one of those cases where the tissues were already in a highly irritable condition, and the irritation set up by that caustic was sufficient to involve the peridental structures to the end of root. In such cases I would wait until the inflammation was allayed, or would assist by the use of local counter-irritants such as tincture of iodine, and after the inflammation had subsided would then use the escharotic if indicated. I have used the aromatic sulphuric acid, but this is also very likely to set up irritation, Robinson's remedy is much better for this as it will not cause irritation; it is made by adding potassium hydrate to carbolic acid until they neutralize each other, this prevents it from being caustic.

I would use caustics in those places where the disease has attacked the bone tissue and created necrosis or caries of bone. In these cases I would not hesitate to use the aromatic sulphuric acid ; I have sometimes used the stronger sulphuric acid in a ten or fifteen percent solution, taking pains, of course, to apply only in the pockets of the teeth. Trichloracetic acid and strong solutions of pyrozone may be used in such cases, as the tissues are in chronic suppuration and not easily influenced or excited.

In regard to the suppurative condition which comes from the gouty diathesis I do not know exactly where I stand, so I am not prepared to say anything in reference to treatment. Shall we resort to systemic treatment or simply attend to local condition ; or shall we send our patients to Florida where they can live on a fruit diet? This question opens up a large field for investigation. Sometimes in these cases I feel as though I do not want to undertake the treatment, as the outcome is so very uncertain, and it is often impossible to do anything for some of the teeth.

Dr. Rix : I want to add that I never undertake to treat these cases until I have removed the inflammation. I do not even remove the deposits until I have accomplished this.

Dr. C. H. Oakman : There is one point in the treatment of pyorrhea which is very interesting to me, and that is the elimination of pain in the process of treatment. While Dr. Hoff was speaking I thought I would bring forth my favorite remedy for all ails, chlorotone, I knew it would tickle him as he has heard of it before from me. I always compare bad pyorrhea cases with the antral troubles, they are both always with me. Once in a while I get a cure, but they are a long time apart.

My success has come from the use of the stronger antiseptics, but those which have not an escharotic power. I do use trichloracetic acid occasionally, and I think there are cases where it is demanded, but I think chlorotone will be used eventually in the treatment of pyorrhea universally. I use it every day one way or another, extracting teeth, etc., but it can be used in these pyorrhea pockets around teeth and all the time you are using it you have the anesthetic as well as antiseptic effect and you can work without discomfort to patient.

Dr. Hoff: How do you use chlorotone, and what for?

Dr. Oakman: I use a saturated solution of chlorotone, I make a hot water solution in a test tube, you can get about a three and a half percent solution in this way. I make it just as hot as the patient can stand it, usually warming the patient up to it by commencing with slightly hot and increasing the temperature gradually. I then saturate a pledget of cotton and press it into the pocket, and by this means I get a very desirable anesthetic effect. I work on two or three teeth at a time in this way. I apply to both labial and lingual of the teeth. Before I dismiss the patient I take a certain number of threads of cotton and saturate in the hot chlorotone solution, and as I place them into place, count them and request the patient to leave them in until the next morning, when they can count them on taking out. A piece of cotton, if left, may act as an irritant, it gets no chance to do so by this method.

There is one question I would ask Dr. Newell, he said that decay is less frequent in teeth attacked with pyorrhea, I would like to ask him if he knows why this is so?

Dr. Newell: I do not know why this is so unless it is due to the formation of some chemical which destroys the lactic acid in the mouth.

In reference to the use of local anesthetics ; I have tried the spray of cocain, but as a rule, I do not find it necessary to relieve pain where I have an abscess cavity, if I cut into the gums extensively I use the cocain solution.

Dr. Essig : I would like to ask Dr. Newell if he has ever used orthoform, it is both an anesthetic and antiseptic, and is very good?

Dr. Newell : I have not. One word in defense of the trichloracetic acid, where we have those rough edges which we find in those red, highly-inflamed odematous conditions ; by the use of this escharotic I get a perfect circle or smooth edge to cavity around the tooth which is very easy to work in. Of course I do not advocate the excessive use of an escharotic. I have no experience in the use of chlorotone as yet.

Dr. S. M. White : I have something which I would like to have you try when you get a case which is real mean to handle. Dry the gums and apply cocain or chlorotone before you commence to remove the tartar, after doing this thoroughly, then apply carefully a twenty-five percent solution of sulphuric acid ; then fill the cavity with quinine and seal it over with chlorotone, and send your patient home to rest and see him again in about a month. I have had very good results from such treatment.

Dr. Hines : There was one point which occured to me while the essayist was reading his paper with which I did not concur, it was in the treatment of the first class of this disease. He advocates the use of a soft rubber brush for the application of the antiseptic mouth-wash which the patient was to use for himself.

I prefer a stiff bristle brush. He referred to the irritation set up. It is my opinion and my experience that a little irritation of this mechanical kind is a good thing, it is a massage. I usually recommend a phrophylactic brush, if it is at first too hard for gums, I request patient to soften it in warm water and as his gums become accustomed to it, increase the stiffness by using colder water. A good mouth-wash for such conditions I think is euthymol one ounce and tannic acid five grains.

Dr. Straith: My experience with pyorrhea has not been very extensive, my first impulse in all such cases has been to send them home and let the disease take its course, still, I have treated a few cases, and of those I attempted, the treatment has been rather successful, although I have used but one mode of treatment and that I heard of in Chicago in the winter of 1895 from Dr. Younger, of San Francisco. He advised a thorough scaling of roots and used simply a ten percent solution of lactic acid. In the treatment of the cases I have attempted, I have added the use of a three percent solution of pyrozone, washing the pockets out several times at one sitting, then drying out and applying the ten percent lactic acid by means of cotton wrapped on a smooth broach. About five or six weeks ago I began the treatment of a very serious case, the patient would have lost the teeth in a few years if they had been neglected. I treated the case just about six times in the manner indicated, and one day last week the patient upon request came in, and much to my surprise the teeth and gums were in a perfectly healthy condition. The teeth were solid where they had been loose before. She expressed herself more than satisfied with the treatment. I could report two other cases where I have used the same treatment with similar success.

I do not care to treat these cases, and there are some I have positively refused to take, but if I think there is any chance I try it.

Dr. Warboys: Since this has resolved itself into a kind of an experience meeting, I can add my word to the discussion. I would like to say that I find the greatest difficulty in the treatment of pyorrhea, I do not seem to possess the required skill necessary to get the tartar off the teeth. If I could get all the tartar off the roots of the teeth and then use thorough disinfection and continue with antiseptic treatment, I would not have much trouble with pyorrhea. Most of us do not possess the skill, or if we do, we do not possess the instruments to remove all the deposits, or, if we possess both of these then it must be we do not possess the patience to do it.

What is the reason we do not get at these deposits? The surfaces involved are obscure and the particles are very small, and it takes great delicacy of touch to remove these particles with the instruments at our command. I do not know but that the instruments are improperly made. It is to me a very difficult operation. I do not know of any other so difficult to me. I succeed best with excavators which are sharp on the side, I pass them around the root of tooth instead of upward or downward. My instrument is sharpened on the side as well as end. After removing calcareous deposits the next thing of course is thorough disinfection. I think it a good plan to leave the pockets open.

Dr. Wise: I think one of the reasons why we do not have more success in the treatment of this disease, is because, when a patient comes in with a case of pyorrhea, we think well this is a case of pyorrhea, I can not do much for it. We get the patient in the chair and touch it up a little and apply some disinfectant and make a charge of fifty cents or a dollar. We do not keep the patient in the chair long enough to treat the case as it should be treated, and make an adequate fee. If we treat the disease as we would any other dental operation and expect a result we will get it. We are simply afraid to charge an adequate fee as should be done.

DR. OAKMAN: I think Dr. Wise has struck the nail on the head. I do not know of any operation in the mouth which should be better paid for.

A patient came to me to have his mouth treated for pyorrhea. He said "A month or two ago I had some work done by a dentist in Chicago who charged me $20.00 an hour for his services." Before I had thought the best thing to do was to extract his teeth but after hearing this I decided the best thing I could do was to attempt a cure, I did, and got excellent results. Although I might have been at first, I was not at all backward in sending the patient a proper bill.

Tell your patients what is the trouble, how bad it is, and explain the treatment which they must undergo.

DR. STRAITH: There is one thing which I would like to add if I may be permitted to speak again, and that is, I use lactic acid to remove all deposits on the roots of teeth, it seems to dissolve them. If you are not able to remove the deposits mechanically, lactic acid will do it.

DR. WISE: In speaking of scraping the roots free from the deposits, I heard one man say he scraped the roots down to their apex, I do not see how he does it, what holds the tooth in? If you do this there is nothing but a hole there and the tooth would fall out. It looks to me as if there was something wrong in some of the arguments here. There is another point, I would like to know why so many dentists depend upon the remedies made by firms when they do not know what is in them. When we studied dentistry at college we also studied a little about medicine and particularly about the medicines we can use in dentistry. Why can not we combine these drugs and get what we want for each case, instead of starting out by using a proprietary remedy. We should make a prescription for each and every case because each case may require something a little different. We should fully diagnose our cases and prescribe proper remedies.

